# Radiopacity of Portland Cement and Calcium Silicate-Based Cement with Different Mixed Ratios of Radiopacifiers

**DOI:** 10.14744/eej.2021.72691

**Published:** 2022-03-28

**Authors:** Cholkamol SARUNKET, Danuchit BANOMYONG, Piyawat VIBULCHAROENKITJA, Supachai SUTIMUNTANAKUL

**Affiliations:** From the Department of Operative Dentistry and Endodontics (C.S., D.B., P.V., S.S. 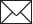 supachai.sut@mahidol.ac.th), Mahidol University, Faculty of Dentistry, Bangkok, Thailand

**Keywords:** Calcium silicate cement, Portland cement, radiopacifiers, radiopacity

## Abstract

**Objective:**

The present study aimed to compare the radiopacity of Portland cement (PC) or calcium silicate-based cement (CSC) with different mixed radiopacifiers [bismuth oxide (BO)/ tantalum oxide (TO) and zirconium oxide (ZO)/barium sulfate (BS)] in a ratio of 1:1 or 1:2, with the 3 mm aluminium thickness (mmAl) acceptable value of ISO 6876:2012.

**Methods:**

PC and CSC mixed with different ratios of radiopacifiers were evaluated. One of high radiopacity radiopacifiers, BO or TO, was mixed with one of low radiopacity radiopacifiers, ZO or BS, in ratio of 1:1 and 1:2. PC or CSC powder, 1.6 g, was added into 0.4 g mixed radiopacifiers. Disc-shaped specimens of 1-mm thickness were prepared by mixing PC or CSC powder containing radiopacifiers with distilled water; the radiopacity was measured according to ISO 6876:2012. One-way ANOVA/Tukey's test and Welch ANOVA/Games-Howell test were used to compare the radiopacity among the groups. The significance level was set at 0.05.

**Results:**

PC groups had a higher radiopacity than CSC groups with the same radiopacifiers and ratio. BO groups showed higher radiopacity than TO groups. The groups with 1:1 ratio provided a higher radiopacity than 1:2 ratio groups (P>0.05).

**Conclusion:**

All tested radiopacifiers revealed adequate radiopacity ranging from 3.05-4.25 mmAl, except CSC with TO/BS in ratio of 1:2.

**HIGHLIGHTS**

• This is the first study measuring the radiopacity of mixed radiopacifiers in Portland cement and calcium silicate-based cement.
• Most of Portland cements and calcium silicate-based cements containing different mixing ratios of radiopacifiers had a radiopacity comparable to the acceptable level of 3 mmAl according to ISO 6876:2012.
• Bismuth oxide is the high-radiopacity radiopacifier that provides higher radiopaque than the others (i.e., tantalum oxide, zirconium oxide, and barium sulfate).

## INTRODUCTION

Portland cement (PC) based material is widely used as root filling materials in endodontic treatments due to its favourable biological properties (1, 2), low solubility, good marginal adaptation, and sealing ability (3). It is composed of tricalcium silicate, dicalcium silicate, tricalcium aluminate and tetracalcium aluminoferrite (4). Metallic impurities such as ferric oxide and aluminum oxide cause the dark grey colour in PC (5). In the manufacturing process of PC, some trace elements such as lead, arsenic, and chromium might be included in the final product that cause undesirable properties and tooth discolouration (6-8). Recently, pure calcium silicate cement (CSC) was developed to substitute PC in calcium silicate-based materials for better biological properties and reduce tooth discoloration induction (9).

Radiopacifier is an important component of calcium silicate-based materials because of the insufficient radiopacity of PC or CSC. The radiopacity values of PC ranged from 0.86-2.02 mm of aluminium thickness (mmAl) (10-13), while pure CSC was 0.99-1.62 mmAl (14, 15). The International Organization for Standardization (ISO) 6876:2012 recommended that radiopacity of root canal sealing material should not be less than 3 mmAl to clearly detect the material on radiograph (16). ProRoot® MTA (Dentsply Tulsa Dental Specialties, Tulsa, OK, USA) was the first PC based material which contained 20% bismuth oxide (BO) as a radiopacifier and provided 5.88-7.5 mmAl radiopacity (15, 17). The radiopacity of element depends on its atomic number (Z), Z of BO is 83 and its radiopacity is high. An element with high Z contains more inner shell electrons that scatter more x-ray photons and cause lesser x-ray photons to expose on receptor, resulting in higher radiopacity (18). However, tooth discolouration associated with the reaction of bismuth oxide has been reported (19, 20). Thus, other alternative radiopacifiers such as tantalum oxide (TO), zirconium oxide (ZO), or barium sulfate (BS) were introduced to replace BO (12, 15, 17, 21).

Another high Z radiopacifier, TO (Z=73) is used in BioAggregate® (Innovative BioCeramix Inc., Vancouver, BC, Canada) with a radiopacity of 5.7 mmAl (22). The TO nanoparticle was studied as a radiopacifier in resin composite and adhesives (23). It is an inert and highly compatible material which enhanced the biocompatibility of other metals through surface nanoparticle coating (24). Nevertheless, TO is a rare earth element, and its high cost limited its cost-effectiveness and clinical use.

The ZO (Z=40) is the radiopacifier of Biodentine (Septodont, St. Maur-des-Fossés, France) and EndoSequence® Root Repair material (Brasseler USA, Savannah, GA: ERRM). With 5% ZO, Biodentine, has a radiopacity of 1.50-2.8 mmAl which resulted from the low Z and less proportion of ZO (25, 26). The radiopacifier in resin composites and endodontic sealers (Epiphany; Pentron, Wallingford, CT, USA), BS (Z=56) have the advantages of white colour, low cost, and high availability (27). The radiopacity values of PC and CSC with 20% BS are only 2.80 and 2.52 mmAl, respectively (12, 14). Increasing the amount of radiopacifying agent to improve the radiopacity is in contrast to the proportion of water to cement ratio and would affect the properties of material (28). To improve the radiopacity of PC and CSC without disturbing their properties, using a combination of low radiopacity radiopacifier with a small ratio of noble high radiopacity radiopacifier might be a choice to improve the radiopacity, resolve tooth discolouration, maintain the desired properties and economic benefit. Thus, the objective of this study was to compare the radiopacity of PC or CSC with different mixed radiopacifiers (BO/TO and ZO/BS) in a ratio of 1:1 or 1:2, with the 3 mmAl acceptable value of ISO 6876:2012. The null hypothesis was the radiopacity of PC or CSC with different mixed radiopacifiers was not significantly different among the groups and to the 3 mmAl level.

## MATERIALS AND METHODS

### Materials preparation

The main ingredient of the experimental materials consisted of 80% PC (SCG, Bangkok, Thailand) or CSC (Alfa Aesar, Thermo Fisher Scientific, Ward Hill, MA, USA), and 20% radiopacifiers, by weight. The radiopacifiers were composed of two radiopacifiers: one with high radiopacity radiopacifier (HRR) – BO (Scharlab, Barcelona, Spain) or TO (Alfa Aesar); and the other with low radiopacity radiopacifier (LRR) – ZO (Riedel-de-Haën®, Loughborough, United Kingdom) or BS (Alfa Aesar). Mixing ratio between HRR and LRR was 1:1 or 1:2. The PC and CSC without radiopacifier were served as control groups (Table 1). Powder of PC, CSC, and radiopacifiers were weighed on the Electronic Analysis Balance (Sartorius, Thailand). The powders were mixed using the geometric dilution method (29) in a glass container of a blending machine [Ultra Micro V-shape Mixer (Tsutsui Scientific Instruments, Tokyo)] until homogeneous.

**TABLE 1.** The mixing ratios and weights of PC or CSC and radiopacifiers (BO/TO and ZO/BS)

Cement powder (g)	Group	Ratios	Radiopacifiers (g)			
			BO	TO	ZO	BS
PC (1.6)	1	1:1	0.2	-	0.2	-
	2		0.2		-	0.2
	3		-	0.2	0.2	-
	4		-	0.2	-	0.2
	5	1:2	0.133	-	0.267	-
	6		0.133	-	-	0.267
	7		-	0.133	0.267	-
	8		-	0.133	-	0.267
CSC (1.6)	1	1:1	0.2	-	0.2	-
	2		0.2		-	0.2
	3		-	0.2	0.2	-
	4		-	0.2	-	0.2
	5	1:2	0.133	-	0.267	-
	6		0.133	-	-	0.267
	7		-	0.133	0.267	-
	8		-	0.133	-	0.267
PC or CSC (2)	control	-	-	-	-	-

PC: Portland cement, CSC: Calcium silicate cement, BO: Bismuth oxide, TO: Tantalum oxide, ZO: Zirconium oxide, BS: Barium sulfate

The main PC composition groups with 1:1 ratio radiopacifiers (PC groups 1-4): 0.2 g of BO or TO, and 0.2 g of ZO or BS, were mixed together for 5 min. Then PC was gradually added to the mixed radiopacifiers and blended for 5 min in a following order: 0.4 g, 0.8 g, and 0.4 g. to obtain 2 g of testing material.

The PC groups with 1:2 ratio radiopacifier (PC groups 5-8): 0.133 g of BO or TO, and 0.267 g of ZO or BS were weighed. The mixing process for this group was the same as in the 1:1 ratio group.

In the CSC groups, the main ingredient was CSC. The weights and mixing procedures of the radiopacifiers and CSC powders were the same as in the PC groups.

### Specimen preparation

**Figure 1.**
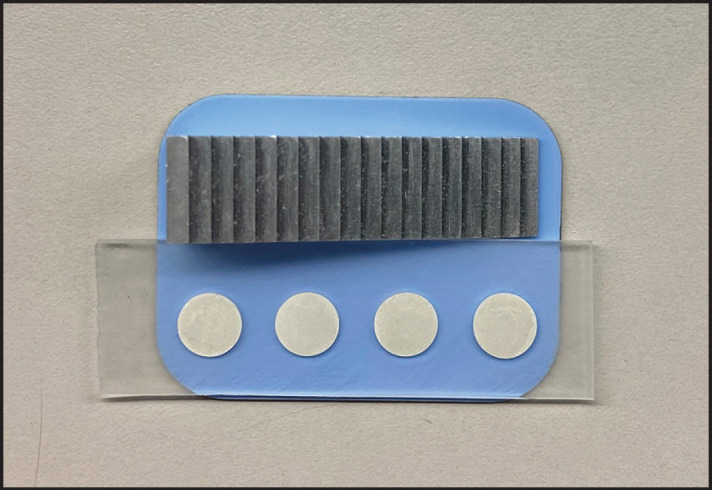
The experimental material discs in transparent acrylic mould and an aluminium step wedge placed on an imaging plate

The disc shape material specimens were prepared according to the ISO 6876:2012 standard for testing dental root canal sealing materials (16). The testing cement powder was mixed with sterile water in a ratio of 2 g powder per 0.7 ml liquid. The mixed material was loaded and condensed into a 6-mm diameter and 1-mm thick acrylic mold which was placed on a glass plate. Another glass plate was placed and pressed on the upper side of the mold to create a flat surface and control the thickness of specimen. The specimens were stored in a moist container at room temperature for 7 days to allow cement setting. The thickness of specimens was checked by a digital caliper (Mitutoyo Corp, Tokyo, Japan). If required, the 320-grit sandpaper was used to polish the specimens to achieve a 1±0.01 mm thickness (Fig. 1). Twelve specimens were prepared for each experimental group.

### Radiopacity assessment

The acrylic mould with 4 specimen discs and an aluminium step wedge was placed on an intraoral photostimulable phosphor (PSP) imaging plate size 2 (Fig. 1). The aluminium step wedge was 98% pure Al with less than 0.01% copper and 1% iron impurities. Its dimensions were 10×36 mm, and the height of steps ranged from 0.5 to 9 mm with 0.5 mm increment.

**Figure 2.**
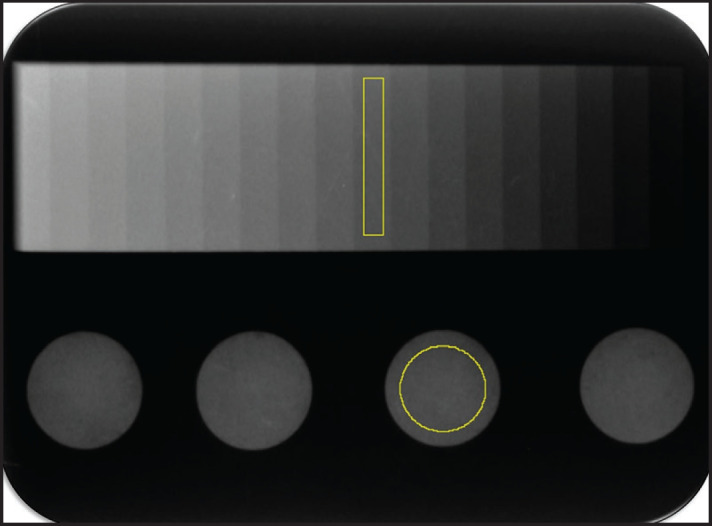
Grey level in radiographic image of material discs and aluminium step wedge in the ImageJ program. The rectangular area on step wedge and circular area on a specimen with the approximate grey level were outlined and the grey levels were calculated for radiopacity value

The X-ray machine (X-mind, Acteon, England) was set at 70 kV, 8 mA, and 0.125 s exposure time, with a fixed 30 cm focus-sensor distance. Three radiographs of each mould were taken repeatedly. The radiographic imaging plate was scanned by an imaging plate scanner (Digora™ Optime, Soredex, Finland), and the images were exported into TIFF files (24 bit). Gray level in each step of the aluminium step wedge and the cement discs were measured by ImageJ software (National Institutes of Health, USA) (Fig. 2). The grey levels of specimens were converted into mmAl by the following equation (14):

**Figure:**
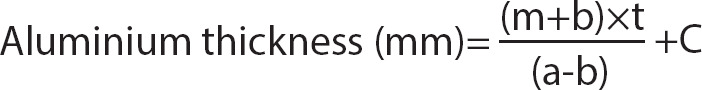


m = grey value of cement disc

b = grey value of the aluminium step wedge below m

a = grey value of the aluminium step wedge above m

t = different thickness between each step of aluminium step wedge which was 0.5 mm

C = mmAl thickness of the step wedge below m.

Grey level of each specimen was measured and averaged from the three radiographs. Average radiopacity (mean±SD) of the experimental materials were compared among the groups and compared with the clinically acceptable 3 mmAl level.

### Statistical analysis

Shapiro-Wilk test showed the normal distribution of data. Levene’s test revealed the homogeneity of variance only in the PC groups, while the heterogeneity was detected in the CSC groups. To compare the radiopacity, One-way ANOVA and Tukey’s test were used for the PC groups while Welch ANOVA and Games-Howell test were used for the CSC groups, with 95% level of confidence. The radiopacity of all groups were compared to the ISO 6876:2012 clinically acceptable level (3 mmAl) with one sample t-test. The significant level of P value was 0.05.

## RESULTS

In control groups without radiopacifier, pure CSC had the lowest radiopacity at 1.00±0.10 mmAl while the radiopacity for pure PC was 1.37±0.14 mmAl. The radiopacity in mmAl of PC groups are shown in Table 2. The radiopacity of PC groups with 1:1 ratio radiopacifiers ranged from the low to high values were as same as the 1:2 ratio groups. With the same pair of radiopacifiers, the 1:1 ratio groups showed higher radiopacity than those of the 1:2 ratio groups.

**TABLE 2.** Means and standard deviations of radiopacity (mmAl) of PC and CSC groups

Radiopacifiers	Ratios	PC groups Radiopacity (mmAl)	CSC groups Radiopacity (mmAl)
-	-	1.37±0.14	1.00±0.10
BO+ZO	1:1	4.25±0.17	4.08±0.25
BO+BS	1:1	4.10±0.18	3.99±0.17
TO+ZO	1:1	3.80±0.18	3.14±0.23
TO+BS	1:1	3.48±0.18	2.93±0.14
BO+ZO	1:2	4.01±0.15	3.50±0.16
BO+BS	1:2	3.88±0.16	3.46±0.36
TO+ZO	1:2	3.76±0.21	3.05±0.18
TO+BS	1:2	3.30±0.27	2.80±0.19

PC: Portland cement, CSC: Calcium silicate cement, BO: Bismuth oxide, BS: Barium sulfate, TO: Tantalum oxide, ZO: Zirconium oxide

**Figure 3.**
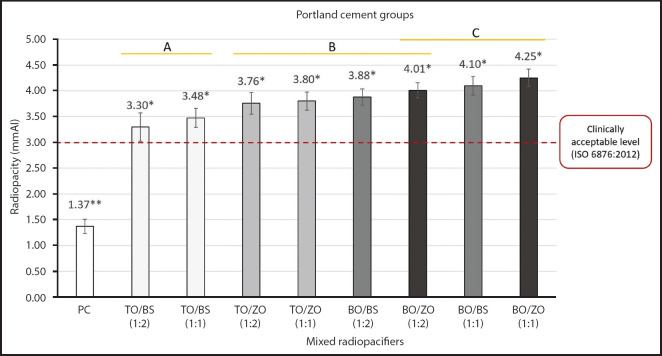
Radiopacity (mmAl) of PC groups with different mixed radiopacifiers and clinically acceptable radiopacity level. Different capital letters indicated a statistically significant difference among the groups (P<0.05) *Indicate a statistically significant difference, higher than the clinically acceptable level (P<0.05), ** Indicate a statistically significant difference, lower than the clinically acceptable level (P<0.05). PC: Portland cement, TO: Tantalum oxide, BS: Barium sulfate, ZO: Zirconium oxide, BO: Bismuth oxide

While comparing the radiopacity within the PC groups, the radiopacity of PC without radiopacifier was significantly lower than other groups (P<0.05). The radiopacity of PC with TO/BS in 1:2 and 1:1 ratio groups was not significantly different, but was significantly lower than the groups of PC with TO/ZO, BO/BS and BO/ZO in both ratios (P<0.05). Radiopacity in groups of PC with TO/ZO (1:2), TO/ZO (1:1), BO/BS (1:2), and BO/ZO (1:2) groups were not significantly different (P>0.05), but TO/ZO (1:2), TO/ZO (1:1) and BO/BS (1:2) were significantly lower than the 1:1 ratio of BO/BS and BO/ZO groups (P<0.05). In groups of BO/ZO (1:2), BO/BS (1:1) and BO/ZO (1:1), the radiopacities were not significantly different (P>0.05) (Fig. 3). 

The radiopacity in mmAl of CSC groups are shown in Table 2. The radiopacity of CSC groups with 1:1 ratio radiopacifiers ranged from the low to high values were as same as the 1:2 ratio groups. With the same pair of radiopacifiers, the 1:1 ratio groups showed higher radiopacity than those of the 1:2 ratio groups.

**Figure 4.**
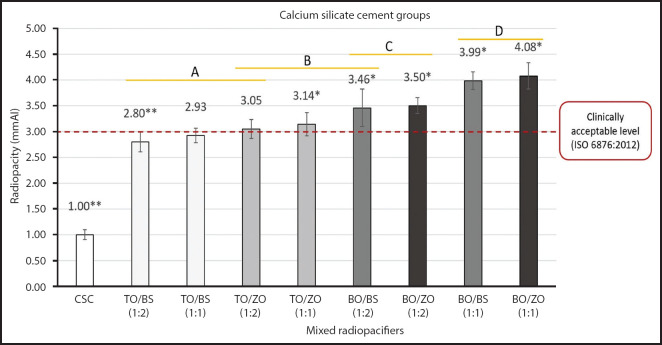
Radiopacity (mmAl) of CSC groups with different mixed radiopacifiers and clinically acceptable radiopacity level. Different capital letters indicated a statistically significant difference among the groups (P<0.05) *Indicate a statistically significant difference, higher than the clinically acceptable level (P<0.05), **Indicate a statistically significant difference, lower than the clinically acceptable level (P<0.05). CSC: Calcium silicate cement, TO: Tantalum oxide, BS: Barium sulfate, ZO: Zirconium oxide, BO: Bismuth oxide

Comparing the radiopacity within the CSC groups, CSC without radiopacifier was significantly lower than other groups (P<0.05). Radiopacity in groups of CSC with TO/BS (1:2), TO/BS (1:1) and TO/ZO (1:2) groups were not significantly different (P>0.05). Both ratios of TO/ZO and 1:2 ratio of BO/BS group were not significantly different (P>0.05). The BO/BS (1:2) and BO/ZO (1:2) groups were not significantly different (P>0.05). The highest radiopacity of CSC was BO/ZO (1:1) which was not significantly different from BO/BS (1:1) (P>0.05) (Fig. 4).

In comparison to the 3 mmAl clinically acceptable level, the radiopacity of PC and CSC groups without radiopacifier were significantly lower than the level of 3 mmAl (P<0.05). The radiopacity of all groups of PC with differentiation of radiopacifiers were significantly higher than 3 mmAl (P<0.05) (Fig. 3). In the CSC groups, the radiopacity of CSC with TO/BS (1:2) group was significantly lower than 3 mm Al, while the radiopacity of TO/BS (1:1) and TO/ZO (1:2) groups were not significantly different (P>0.05). The radiopacity of CSC with TO/ZO (1:1) group and both ratio of BO/BS and BO/ZO groups were significantly higher than 3 mmAl (P<0.05) (Fig. 4).

## DISCUSSION

The null hypothesis of this study was rejected as there were statistically significant differences among the radiopacity in the PC or CSC groups with different mixed radiopacifiers, and to the 3 mmAl acceptable level. The radiopacity of the PC groups and CSC groups reacted in the same manner. When comparing the groups with same radiopacifiers and same ratios, PC groups had a higher radiopacity than the CSC groups. This might be due to the difference in compositions of PC and CSC. The CSC powder was composed of only tricalcium silicate and dicalcium silicate, whereas PC contained more components apart from tricalcium silicate and dicalcium silicate, such as tricalcium aluminate, tetracalcium aluminoferrite and other, more metallic substances that provided higher level of radiopacity (4).

The PC and CSC in this study had low radiopacity at 1.37±0.14 and 1.00±0.10 mmAl, respectively, which were in the same range as reported in previous studies (14, 15). These radiopacities were not different from the 1.74±0.02 mmAl of root dentine (12). Therefore, a radiopacifying agent was necessary for PC and CSC to distinguish from root dentine. The BO was the first radiopacifier with a high radiopacity used in CSC. Previous studies reported the adequate radiopacity of PC with 20% weight of BO ranged between 5.88-7.5 mmAl (15, 17). The high radiopacity of BO related to its high atomic number (Z=83) which tended to absorb or scatter a large amount of x-ray attenuation. However, many studies reported tooth discolouration induction associated with BO (19, 20). Another radiopacifier with a high atomic number, TO (Z=73) possessed as an alternative to replace BO. Because of TO is a rare earth element with an extremely high cost, a combination of high and low radiopacity radiopacifiers served as an option to reduce the amount of TO and the manufacturing cost. The right ratio of radiopacifiers could still maintain the adequate radiopacity of more than 3 mmAl. 

The radiopacifiers in this study were divided into two groups according to the Z value of elements in compound, including HRR (BO and TO) and LRR (ZO and BS) that were mixed in a ratio of 1:1 or 1:2. The experimental groups that contained BO provided higher radiopacity than those with TO, corresponding to their atomic numbers. On the contrary, in the groups with same HRR and ratio, ZO groups showed higher radiopacity than BS groups. The atomic number of ZO was 40 and was lower than that of BS, which was 56. This might be due to the difference in the density-related structure of ZO and BS molecules. Theoretically, one-unit volume of ZO was 56% smaller than that of BS that caused the closer inner shell electrons in ZO structure which increase the ability of absorbing or scattering x-ray photon and caused higher radiopacity (30).

Corresponding to the atomic number, the radiopacity of TO/ZO groups were higher than TO/BS groups regardless of the ratio. The 1:1 ratio BO/BS group showed a higher radiopacity than 1:2 ratio BO/ZO group. It may be that BO was highly radiopaque and a greater amount of BO in the ratio 1:1 group acted dominantly on radiopacity. In the groups of PC or CSC with the same type of main ingredient and radiopacifiers, the 1:1 ratio of HRR/LRR groups always showed the higher radiopacity than that with 1:2 ratio. This could simply be explained by the greater amount of HRR in the groups with 1:1 ratio. 

Most of the studies of PC and CSC reported the radiopacity with a single radiopacifier. Vibulcharoenkitja et al.,(14) reported that the radiopacity of CSC with an addition of 20% BO was 5.42±018 mmAl. Comparing this to the results in our study, the CSC with 10% BO (HRR/LRR ratio 1:1) and 6.33% BO (HRR/LRR ratio 1:2) groups provided the radiopacity at 3.99-4.08 and 3.46-3.5 mmAl, respectively. Even when the ratios of BO were reduced, the radiopacities were still higher than the clinically acceptable value. The radiopacity of CSC with 20% TO was reported at 3.39±0.10 mmAl (14). The ratios of TO in CSC were reduced to 10% and 6.33% in this study that resulted in radiopacities in a range of 2.93-3.14 and 2.8-3.05 mmAl, respectively, which were lower than the clinically acceptable value. Therefore, the results showed that BO provided higher radiopacity than TO.

Most of the materials with different radiopacifiers and ratios in this study showed adequate radiopacity above the 3 mmAl acceptable level, which could be clinically detected in the radiograph and easily distinguished from the root dentine (12). Only the group of CSC with 1:2 ratio TO/BS was significantly lower than the acceptable value. This exceptional low radiopacity may be due to the combination of low radiopacity CSC, the inferior radiopacity of TO and BS, and the reduced amount of TO. This was an initial study. The data and references were limited. The result was only from laboratory study that could not be clinical relevant. Many clinical factors might involved in the properties of materials. Further study must be evaluated in various aspects.

## CONCLUSION

All groups of PC and CSC with different mixing ratio of radiopacifiers had a radiopacity comparable to the acceptable level of 3 mmAl according to ISO 6876:2012, except the group of CSC with TO/BS in 1:2 ratio. The BO groups with any ratios and LRR provided a higher radiopacity than the TO groups. The BO groups in 1:1 ratio of any LRR provided higher radiopacity than other groups.
